# Correlation between measles vaccine doses: implications for the maintenance of elimination

**DOI:** 10.1017/S0950268817003077

**Published:** 2018-02-21

**Authors:** A. McKee, M. J. Ferrari, K. Shea

**Affiliations:** Department of Biology, The Pennsylvania State University, 208 Mueller Laboratory, University Park, PA 16802, USA

**Keywords:** Disease elimination, measles, modelling, routine immunisation, vaccine policy

## Abstract

Measles eradication efforts have been successful at achieving elimination in many countries worldwide. Such countries actively work to maintain this elimination by continuing to improve coverage of two routine doses of measles vaccine following measles elimination. While improving measles vaccine coverage is always beneficial, we show, using a steady-state analysis of a dynamical model, that the correlation between populations receiving the first and second routine dose also has a significant impact on the population immunity achieved by a specified combination of first and second dose coverage. If the second dose is administered to people independently of whether they had the first dose, high second-dose coverage improves the proportion of the population receiving at least one dose, and will have a large effect on population immunity. If the second dose is administered only to people who have had the first dose, high second-dose coverage reduces the rate of primary vaccine failure, but does not reach people who missed the first dose; this will therefore have a relatively small effect on population immunity. When doses are administered dependently, and assuming the first dose has higher coverage, increasing the coverage of the first dose has a larger impact on population immunity than does increasing the coverage of the second. Correlation between vaccine doses has a significant impact on the level of population immunity maintained by current vaccination coverage, potentially outweighing the effects of age structure and, in some cases, recent improvements in vaccine coverage. It is therefore important to understand the correlation between vaccine doses as such correlation may have a large impact on the effectiveness of measles vaccination strategies.

## Introduction

As of early 2017 measles was officially eliminated from seven countries in the Western Pacific, 24 countries in Europe, and all countries in the Americas, despite occasional outbreaks that threaten this elimination status [1–3]. Measles is a leading cause of vaccine-preventable childhood death [4], and maintaining successful elimination at the country-level is key to regional elimination goals [5, 6]. Due to increased globalisation [7] and the highly transmissible nature of the disease, measles is at constant risk of re-emergence in the countries where it is eliminated. Several resurgent outbreaks have occurred; for example, the 2013 outbreak in Brazil, which lasted more than 1 year and changed Brazil's elimination status [1, 8], the 2015 outbreak in Mongolia [9], which occurred less than 1 year after Mongolia was certified measles free [10], and the 2017 outbreak in Romania, which threatens much of Europe's elimination status [3]. A re-evaluation of management strategies for measles in order to better maintain elimination would be valuable [11].

High levels of immunity within the population are critical to maintain elimination. When immunity is high, outbreaks caused by reintroduction events have lower incidence and shorter duration [12]. As measles is a particularly highly transmissible disease, the herd immunity threshold (that is, the proportion of the population that needs to be immune in order to prevent a reintroduction event sparking prolonged re-emergence of the disease) is commonly considered to be about 95% [13]. The true threshold in any location depends on population structure [14], and is difficult to measure directly; however, maximising the proportion of the population that is immune is the best way to ensure that that proportion is above the true threshold. In order to maintain high levels of immunity, extensive vaccination efforts are continued in countries that have achieved elimination. In most countries, these vaccination efforts involve administering two doses of measles-containing vaccine (MCV) [6, 15].

**Fig. 1.**
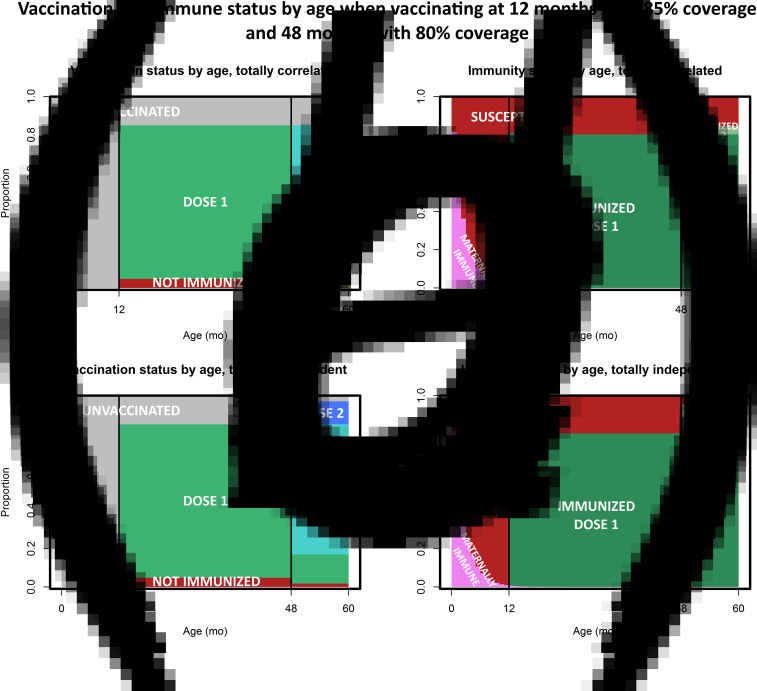
Vaccination and immunity class breakdown when first-dose coverage is 85% and second-dose coverage is 80%. (a) The vaccination classes when doses are administered dependently. The rate of primary vaccine failure (the height of the red section at the right edge of the panel) is 0.6%. (b) The immune classes when doses are administered dependently. The proportion of the population left susceptible (the total proportional size of the red portion over the entire population, not just 0–60 months as shown here), is 18.9%. (c) The vaccine classes when doses are administered independently. The rate of primary vaccine failure (the height of the red portion at the right edge of the panel) is 1.8%, which is greater than when doses are administered dependently. (d) The immune classes when doses are administered independently. The proportion of the population left susceptible (the total proportional size of the red portion over the entire population) is 10%, which is less than when doses are administered dependently.

Typically, health agencies are concerned with maximising the coverage of each dose [16–18]; that is, the proportion of the population that gets vaccinated at each specified target age. However, coverage alone does not tell the full story. The proportion of the population that is immune to measles differs from, but directly depends on, the proportion of the population that gets at least one dose of MCV. The reason for this difference is twofold: first, maternal immunity interferes with the efficacy of the vaccine [19], and second, the fraction of the population below the age of first vaccination limits the maximum possible population immunity [20]. There are three subparts of the susceptible population maintained by a two-dose strategy (Fig. 1, the red part in panels (b) and (d)): (1) individuals who are too young to receive the first dose (the portion of the red part left of the left black line), (2) individuals who missed the first dose or for whom the first dose was ineffective but are too young to receive the second dose (the red part between the two black lines), and (3) individuals who either missed both doses or who failed to seroconvert after receiving one or both doses (the portion of the red part to the right of the right black line). These subparts (not bounded to a limited age range, as in the figure) sum to the total proportion of the population that is susceptible, and can contribute to transmission in case of a reintroduction event. Minimisation of the total proportion of the population that is susceptible requires judicious targeting of vaccination age targets bearing in mind all three subparts [11].

To maximise immunity, the first dose of MCV(MCV1) should be administered early enough to minimise the number of susceptible individuals who are too young to have yet received a vaccine, but late enough that maternal-acquired antibodies, which interfere with the efficacy of the measles vaccine, have sufficiently waned, so that relatively few individuals fail to seroconvert. The second dose (MCV2) is administered later in life, with two purposes: one, to catch children for whom the first dose was not effective, and two, to provide a second opportunity for children who did not have the first dose to receive at least one dose. In most countries, the first dose of the measles vaccine is administered when the child is 9–12 months old, and the second dose is administered sometime later in life, often immediately before school entry [15]. The effectiveness of this strategy directly depends on the coverage of each dose [10]. However, as we illustrate below, it also depends on the likelihood of an individual receiving a second dose, conditional on their having received the first.

If the second dose of MCV acts as a true second dose for all individuals – that is, it is the second dose an individual receives in their life – then the population receiving the second dose is a nested subset within that receiving the first dose. We refer to that as ‘perfect correlation’ in this paper. However, if all children are equally likely to receive a dose at the second age target, regardless of whether they had the first dose, then the second dose acts as a second opportunity to be provided with at least one dose. Formally, then, the populations receiving each dose are independent (correlation between doses is 0). Practically, the distribution of the second dose may vary between these two extreme scenarios; that is, the correlation may range between 0 and 1. In principle, second-dose vaccination programmes may preferentially target children who did not receive the first dose; in our model, we would call that negative correlation. However, due to correlations in healthcare access and demand, this is a rare occurrence in the real world [21], as such we do not address this formally here, but return to these programs in the discussion.

Understanding the effects of the correlation between the populations receiving the first and second doses of MCV (hereafter referred to as just ‘correlation’) on population immunity resulting from a two-dose strategy can provide two key insights. It can provide a more accurate estimate of the range of population immunity maintainable by a two-dose strategy with specific coverages, illustrating the benefit of designing a surveillance system to monitor correlation. It can also suggest ways to adapt management strategies, here the vaccine schedule, to account for the observed correlation. To do this, we model the effects of correlation on the maintainability of measles elimination using an age-structured model for the distribution of immunity within a population in the absence of disease. We use this model to find the proportion of susceptible individuals remaining in the population when vaccinating with specified coverage at specified first- and second-dose age targets, with the specified correlation between the populations receiving each dose. We also find the proportion of individuals who have received at least one dose, but have not seroconverted (i.e. primary vaccine failure). Finally, we consider the interaction between coverage and correlation for a range of age structures.

## Methods

We use an age-structured model, based on an earlier two-dose measles model [11], to algorithmically determine the proportion of individuals left susceptible by a given vaccine schedule in the absence of disease (i.e. in the elimination setting). We divide age classes monthly up to 5 years, and yearly afterward, until 75 years of age. From a specified coverage and age target for each dose, as well as specified correlation, we divide each age class into four vaccine classes (Fig. 1a and c); unvaccinated (shown in grey), a recipient of the first dose only (shown in green), a recipient of the second dose only (shown in dark blue) and a recipient of both doses (shown in light blue). Using an age-specific efficacy function (based on an age-specific probability of retaining maternal immunity; see *p*_mT_ below), we then divide each vaccine class into three immunity classes; susceptible, maternally immune, and effectively immunised. We then calculate the age distribution and proportion of susceptible individuals left in the population by multiplying the age-specific probability of being susceptible with the age structure and compare across multiple age structures. We also find the rate of primary vaccine failure from the proportion of susceptible individuals who received at least one dose.

Children born to immune mothers are born with maternal immunity, which interferes with the efficacy of the vaccine. Therefore, the proportion of children who seroconvert due to vaccination depends on the proportion who were born with maternal immunity. The proportion of children who were born with maternal immunity depends on the proportion of their mothers who are immune, which in turn depends on the proportion of their mothers who were born with maternal immunity. Our dynamical model accounts for these generational shifts in maternal immunity and resulting vaccine efficacy, and can account for changes in vaccination coverage (Supplementary Material 1). However, for simplicity, we assume the system is at equilibrium, so that vaccination coverage is constant and the proportion of children born with maternal immunity is constant and equal to the proportion of adults that have been effectively immunised by vaccination. Notably, we omit any infection-derived immunity as we are concerned with the maintenance of elimination in the disease-free setting. Disease could be included in an extension of our model to other settings, but disease only increases immunity, so the disease-free setting will always provide a conservative estimate of the actual immunity maintained.

Let *v*_1_ be the coverage of the first dose, and *v*_2_ be the coverage of the second dose. The proportion of children who get at least one dose, assuming *v*_1_ ⩾ *v*_2_, is bounded by the coverage of the first dose, *v*_1_ (which is the case when everyone who receives the first dose receives the second dose), and either 100% or the sum of the coverages of each dose, *v*_1_ + *v*_2_ (when the second dose is administered first to people who did not receive the first dose). If the interaction between the populations receiving each dose is at least independent, the population receiving at least one dose is bounded by *v*_1_ and 1 − (1 − *v*_1_) × (1 − *v*_2_). We describe this interaction between the populations receiving the first and the second doses as correlation. For our purposes, we define the correlation between vaccine doses to be the proportion of the second-dose administered non-independently to people who receive the first dose. When the correlation is one, the second dose is administered solely to children who had the first dose, achieving only the aim of providing those individuals a second chance to become immune in case of primary vaccine failure. When the correlation is zero, the second dose is administered independently of whether the child had the first dose – notably, in this case, the ‘second dose’ means only a dose administered at the second target age, not necessarily the second dose a specific individual receives. When doses are independent, all individuals have a second chance to be vaccinated, regardless of whether they have been vaccinated already.

To divide age classes into vaccination classes, we assume a proportion of individuals equal to the coverage of the first dose, *v*_1_, is vaccinated with the first dose at the first age target, *t*_1_, and therefore moves from the unvaccinated class into the first-dose only vaccine class. This proportion remains constant up to the age target for the second dose, *t*_2_, when a proportion of individuals are vaccinated with the second dose. For the purposes of this paper, we assume the first-dose age target is 12 months and the second-dose age target is 48 months, although the qualitative results are the same regardless of age targets chosen.

Let *corr* be the correlation between the two doses. If *v*_1_ ⩾ *v*_2_, then *p*(*MCV*2|*MCV*1) = *v*_2_(1 − *corr*) + (*v*_2_/*v*_1_)*corr*. A proportion, 1 − *corr*, of the second dose is administered independently to people who received the first dose, such that the proportion *v*_2_(1 − *corr*) of people who had the first dose independently receive the second. A proportion, *corr*, of people who had the first dose receive the second non-independently. Since coverage of the second dose is constant, regardless of correlation, this means that a proportion (*v*_2_/*v*_1_)*corr* of people who had the first dose receive the second non-independently. We can similarly find the proportion of people who receive the second dose given that they did not have the first dose: *p*(*MCV*2 | ¬*MCV*1) = *v*_2_(1 − *corr*), as only 1 − *corr* doses are administered independently to people who did not receive the first dose. Here, | means ‘given’ and ¬ means ‘not’, so *p*(*MCV*2 | ¬*MCV*1) is the proportion that get MCV2 given that they did not receive MCV1. Therefore, 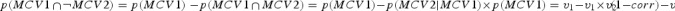 adults receive only the first dose, 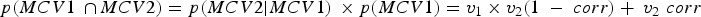 adults receive both doses, and 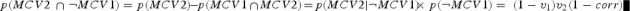 adults receive only the second dose. The remainder is unvaccinated. We can obtain similar equations for when *v*_1_ < *v*_2_ (Supplementary Material 2).

Not all doses of vaccine successfully confer immunity in the recipient. A primary reason for this failure to seroconvert is interference of maternally derived antibodies [19], although we also include the chance that the vaccine was rendered ineffective by other means, such as cold-chain failure [22]. To divide vaccine classes into immune classes, we developed a function for age-specific efficacy based on maternal immunity. The proportion of individuals born with maternal immunity in generation T, *p*_*mT*_, is the proportion of individuals successfully immunised in the previous generation [11, 20]. Assuming the coverage of the first dose exceeds the coverage of the second, coverage is unchanging over time, and each gender is vaccinated equally:

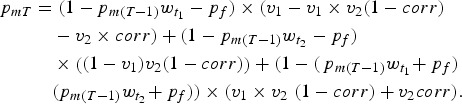


We can then solve for the equilibrium value of *p*_*m*_ using the quadratic formula [11]. Here, *w*_*t*_ is the waning function for maternal immunity, so 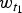 is the proportion of individuals born with maternal immunity that retain immunity at the first target age, and 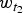 is the proportion of individuals born with maternal immunity that retain it at the second target age. We assume that this waning function is exponential with a mean equal to 3 months [23]. We assume *p*_*f*_, which is the probability that the vaccine fails for some other reason, such as cold chain failure, is constant across age classes – specifically, we assume a constant 5% failure rate for reasons not relating to the immune status of the recipient [22]. We also assume that vaccine failure is independent at each dose; that is, the probability that the second dose an individual receives fails does not depend on whether they received a first dose which failed.

To find the rate of primary vaccine failure, we simply take the difference in the proportion of adults who have had at least one dose and the proportion of adults who are immune. This rate depends on both the waning of maternal immunity and the correlation between doses.

## Results

**Fig. 2.**
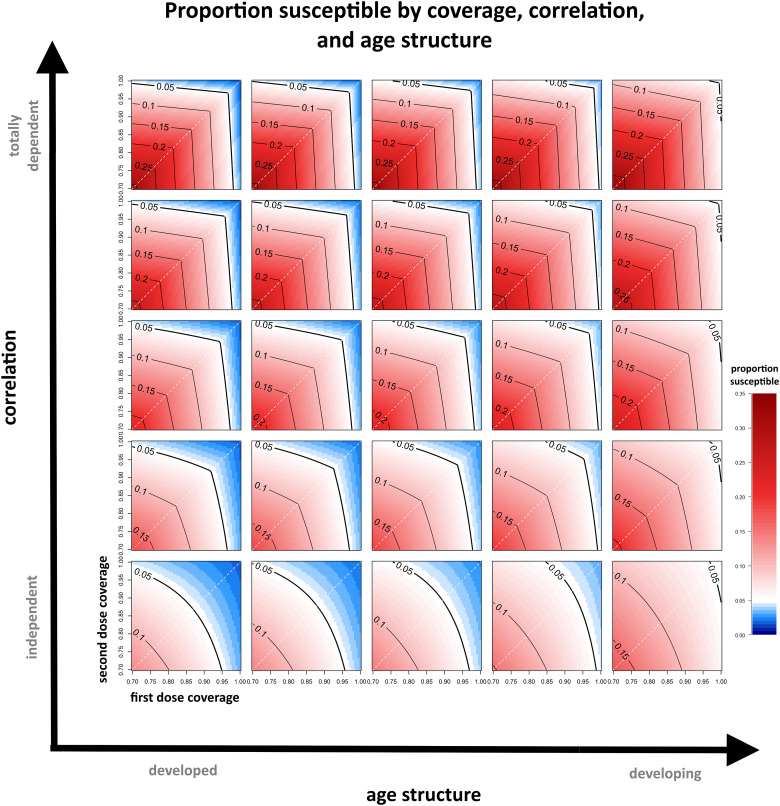
The susceptible proportion remaining for a range of first- and second-dose coverages, correlations and age structure parameters. The contours indicate various threshold levels of immunity, where <5% susceptible within the population is generally considered sufficient to maintain elimination and is coloured in blue. The white dashed line indicates where the coverage of each dose is equal.

For a constant level of first- and second-dose coverages, reducing the correlation between doses increases the proportion of the population that receives at least one dose, but decreases the proportion of the population that receives both doses (Fig. 1), increasing population immunity. Individuals who received a dose at the first age target but did not seroconvert are less likely to receive a dose at the second age target when correlation is low, and so are less likely to be given a second chance to seroconvert, increasing the rate of primary vaccine failure (Fig. 1b and d). Improving coverage of either dose will always reduce the proportion of individuals that are susceptible, regardless of correlation. However, improving coverage is not the only way to reduce the susceptible proportion – reducing correlation does so as well (Fig. 2). This holds true regardless of the underlying age structure.

When doses are administered independently, a relatively large proportion of the population gets at least one dose (Fig. 1), and increasing the coverage of either dose has a large effect on population immunity (Fig. 2). For example, if the first dose has 80% coverage and the second dose has 50% coverage, and the doses are administered *independently*, then the second dose is administered with equal proportional coverage to individuals who received the first dose and individuals who missed it. In this case, 90% of the population receives at least one dose. Improving second dose coverage by 10% would provide an additional 10% coverage to the population of individuals who missed the first dose; 92% of the population would receive at least one dose. In comparison, improving first dose coverage by 10% would provide an additional 10% coverage to the population of individuals who will later miss the second dose; this is a larger population, and 95% of the total population would receive at least one dose. Regardless, improving coverage of either dose increases the proportion of the population that receives at least one dose and reduces primary vaccine failure.

In contrast, high correlation reduces the benefit incurred by increasing the coverage of a given dose. When doses are administered in a totally dependent fashion, increasing the coverage of the dose with lower coverage has very little benefit. For example, if the first dose has 80% coverage and the second dose has 50% coverage, and the doses are *totally dependent*, then the second dose is administered only to individuals who have already had the first dose; 80% of the population receives at least one dose. Increasing the coverage of the second dose by 10% only increases the proportion of the population receiving both doses, and therefore reduces primary vaccine failure, but will not increase the proportion of the population receiving at least one dose. By comparison, increasing the coverage of the first dose by 10% will increase the proportion of the population receiving at least one dose to 90% and thus have a much larger effect on the resulting population immunity. However, this will increase the proportion of people who get just one dose, and therefore increases primary vaccine failure.

**Fig. 3.**
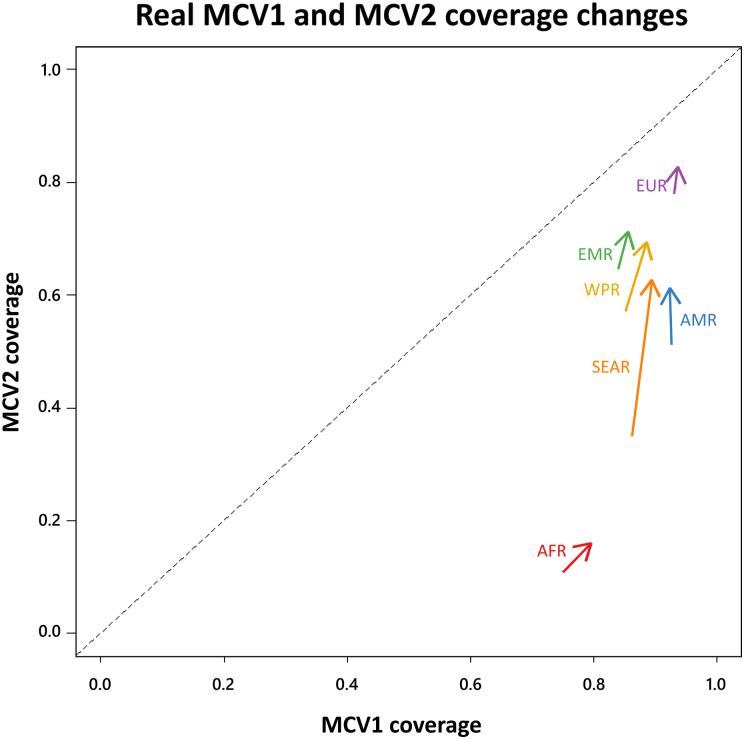
The average reported MCV1 (*x*-axis) and MCV2 (*y*-axis) coverages for each of six WHO health regions between 2005 and 2014. The unpointed end of the arrow represents the average coverage from 2005 to 2009. The pointed end represents the average coverage from 2010 to 2014. Missing reports were assumed to be 0. AFR is the African Region, AMR is the American Region, EMR is the Eastern Mediterranean Region, EUR is the European Region, WPR is the Western Pacific Region, and SEAR is the Southeast Asian Region.

**Fig. 4.**
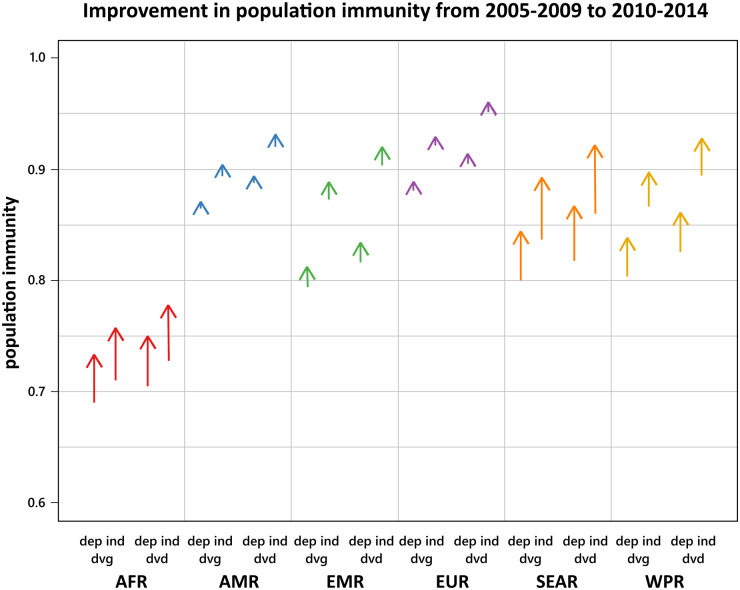
The improvement in population immunity for each WHO health region from 2005–2014, in each of four different contexts, using coverages shown in Figure 3. AFR is the African Region, AMR is the American Region, EMR is the Eastern Mediterranean Region, EUR is the European Region, WPR is the Western Pacific Region, and SEAR is the Southeast Asian Region. The unpointed end is the population immunity maintained by the average first- and second-dose coverages from 2005 to 2009, and the pointed end is the population immunity maintained by the average first- and second-dose coverages from 2010 to 2014. The four contexts are the four crosses of developing and developed age structures (‘dvg’ and ‘dvd’, respectively) with dependent and independent administration of doses (‘dep’ and ‘ind’, respectively). Each of these contexts represents a corner of Figure 2.

It is important that we know the correlation between doses when estimating population immunity from vaccine coverage. Second-dose coverage has improved from 2005 to 2015 in all six WHO Health regions, and first-dose coverage has improved in five of them (all regions except the Americas (AMR)) (Fig. 3). To illustrate the interaction of coverage, correlation, and population structure, we estimated population immunity from those coverages for four contexts: (i) developing age structure with doses administered dependently, (ii) developing age structure with doses administered independently, (iii) developed age structure with doses administered dependently and (iv) developed age structure with doses administered independently (Fig. 4). The two age structures differ in terms of the proportion of children under five (this proportion is higher in the “developing” setting). Each region is made up of many countries, all with different age structures, so we compared the population immunity for the two extremes of the range of age structures, rather than estimating the true average age structure of a region. For each health region, the level of population immunity maintained by vaccination was much greater when doses were administered independently. In four health regions (the American Region (AMR), the Eastern Mediterranean Region (EMR), the European Region (EUR) and the Western Pacific Region (WPR)), the improvement in population immunity achieved by the improvement in coverage was dwarfed by the improvement in population immunity that could be achieved by completely decorrelating the first and second doses. The Southeast Asian Region (SEAR) had a much larger increase in coverage than the others (Fig. 3), so coverage improved population immunity slightly more than completely decorrelating doses would (Fig. 4). In the African Region (AFR), first-dose coverage improved a lot relative to second-dose coverage (Fig. 3), so the improvement in coverage significantly improved population immunity (Fig. 4). In all regions, completely decorrelating first and second doses would have a larger effect on population immunity than differences in age structure do. In regions close to the elimination threshold, the correlation between the doses could be the difference between achieving (or maintaining) elimination or not, although this will depend on the specific local age structure [11].

## Discussion

In most countries, a two-dose routine immunisation strategy is used [15] to maintain high levels of population immunity to measles and, in places where the disease is eliminated, prevent prolonged re-emergence of this deadly disease [1]. The second dose of a two-dose strategy to maintain measles elimination is administered with two main goals [24]. One is to provide a second chance for individuals for whom the first dose was ineffective to become immune. The second is to provide a chance for individuals who missed the first dose to be vaccinated at all. Correlation between the two doses affects how well each of these aims is met, with high correlation improving performance under the first objective and low correlation improving performance under the second objective. When coverage of at least one dose is 100%, then everyone receives at least one dose, regardless of correlation. However, when coverage falls short of 100%, the correlation has a significant impact on the proportion of the population receiving at least one dose, and therefore on the proportion of the population that is immune, with lower correlation resulting in higher population immunity, despite higher primary vaccine failure.

Correlation is unlikely to be independent of age structure, coverage or health system; it is possible to speculate about relevant issues. Deficiencies in coverage tend to arise from two sources; lack of vaccine demand and poor healthcare access [25, 26]. As vaccine demand and healthcare access depend on socioeconomic factors that are highly heterogeneous in a population [27, 28], it is likely that correlation between populations receiving the first and second doses is high in the absence of programmes specifically designed to address these heterogeneities. Deficiencies in coverage can then be addressed with a combination of demand creation programmes and efforts to improve healthcare access, via appropriately tailored communications strategies [29]. Conventionally, these efforts might be focused on improving coverage; our work suggests that changing correlation might be another productive target, and that focusing only on the coverage of one dose might be beneficial if it catches people who otherwise would not be vaccinated at all. While not reported explicitly, correlation can be inferred in real situations from reports of vaccine ‘completeness’ and coverage of fully immunised children [30–32]. However, such reports often do not report the relative size of the partially vaccinated or completely unvaccinated populations.

The level of correlation between doses can have significant implications for the performance of vaccine schedules and strategies to improve population immunity. In two-dose schedules, it may seem intuitive to focus on improving coverage of the dose that has the lower coverage. However, if the correlation between doses is high, this will, in fact, be less beneficial than improving coverage of the higher coverage dose. Average MCV2 coverage was lower than average MCV1 coverage in every WHO health region between 2005 and 2014 (Fig. 3 – all arrows fall below the 1 : 1 line). If doses in the component countries were administered dependently, then focusing on increasing first-dose coverage would have the biggest impact on population immunity; however, MCV2 coverage increased far more than MCV1 coverage did in all regions except Africa, in fact, in the American health region, average MCV1 coverage actually declined while MCV2 coverage increased. While high MCV2 coverage is important to effectively maintain elimination, its importance relative to maintaining high MCV1 coverage depends on correlation.

The effects of correlation are likely to pertain to supplemental immunisation activities (SIAs) as well as routine immunisation. SIAs are typically thought to be able to reach populations that are not reached by the routine health system [33], that is, independent of routine coverage, but that may not always be the case [34]. If the likelihood of being vaccinated during an SIA is independent of routine vaccination, then SIAs will substantially improve the proportion of the population receiving at least one dose. However, SIAs are logistically difficult to perform, and may reach the populations with the best access to the health system first [28], meaning that they may be administered primarily to children who also receive routine immunisation. If the populations reached by SIAs are highly correlated with the populations receiving routine immunisations, the true impact of SIAs could be greatly overestimated, leading to misconceptions about the true state of immunity in the population unless we account for correlation. Some simulation studies have been done to illustrate this paradigm for outbreak response vaccination [35].

A high correlation may result from routinely missed populations (e.g. [21, 36–38]) and heterogeneities in healthcare access that result in positive feedback mechanisms, e.g. poverty traps [39]. While increasing the coverage of either dose always decreases the proportion left susceptible in the population, exploring novel vaccination strategies may provide additional opportunities to reduce the susceptible population without necessarily improving coverage of either dose. Vaccination strategies or targeted communication strategies could be adapted to reduce correlation, by specifically targeting vaccine-hesitant groups or groups with poor healthcare access [40, 41]. Ideally, these strategies would achieve negative correlation, where most children receiving the second dose did not receive the first dose. For example, mobile vaccination strategies could provide opportunities for groups with poor healthcare access to receive at least one dose [42]. Accounting for correlation among doses when modelling the spatial distribution of non-vaccination [43], and incorporating local transmission and mixing patterns, could provide estimates of how much population immunity would be improved by second-dose strategies. Formal accounting of how proposed strategies reach people who are not otherwise reached by the routine health system remains a critical area of operational research [44–46]. Communication strategies may be a necessary part of decreasing correlation by increasing vaccine demand; they were vital in the early years of polio eradication efforts [41].

Decorrelating doses does come at the cost of vaccine effectiveness; as fewer individuals receive two doses, fewer individuals receive both opportunities to seroconvert. This may cause misleading indicators, as the observed rate of primary vaccine failure will increase, perhaps indicating a failure of vaccination in a place where the susceptible proportion is actually decreasing. While our model is relatively simple, a more complex model could explore this trade-off in full.

The simplicity of our modelling approach comes with one significant advantage, however, in that it could be applied to many diseases with a multiple dose vaccine schedule and potentially expanded to account for correlations in vaccine failure. The trade-off between primary vaccine failure and the proportion of the population receiving at least one dose that comes with correlation pertains to many other vaccine schedules. However, if individuals require multiple doses to successfully seroconvert, population immunity may depend more on the proportion of the population that receives at least two doses than on the proportion of the population that receives at least one. In this case, the high correlation would outperform low correlation with respect to population immunity. Thus, the relative benefits of low or high correlation could be reversed for other vaccines, depending on the probability that an individual fully seroconverts following just one dose. In short, our work shows that careful consideration of both coverage and correlation between doses of vaccine may allow improvements in population immunity that would be more difficult to achieve by addressing coverage alone.

## Supplementary material

For supplementary material accompanying this paper visit https://doi.org/10.1017/S0950268817003077. click here to view supplementary material
