# Supplementary material for: Correlation between measles vaccine doses: implications for the maintenance of elimination
Source: Epidemiol Infect. 2018 Feb 21;146(4):468–75. doi: 10.1017/S0950268817003077 (PMC5848754; doi:10.1017/S0950268817003077)
Supplement: Supplementary file 1 [file S0950268817003077sup001.zip › S0950268817003077sup001/Correlation_Supplement.1_10.2.2017.docx]

To find the age distribution of immunity following vaccination, we use the following function:

firstSecond <- function(cov1st, cov2nd, corr2nd, target1st, target2nd, pop, eff, failure){

#cov1st is 1st dose coverage – a single value between 0 and 1

#cov2nd is second dose ocoverage – a single value between 0 and 1

#corr2nd is the correlation between the 1st and 2nd doses#target1st is the first dose age target – a single value between 0 and 1

#target1st is the first dose age target – an index value corresponding to the index of the age class where vaccination happens

#target2nd is the second dose age target – an index value corresponding to the index of the age class where vaccination happens

#pop is the age structure – a vector

#eff is the age specific efficacy due to interference by maternal antibodies – a vector of the same length as pop

#failure is the constant failure rate due to other factors (e.g. coldchain failure) – a single value between 0 and 1

###ACCOUNTING

#figure out what proportion get both the first and second dose

if(cov1st >= cov2nd){

firstSecond = cov1st*(cov2nd*(1 - corr2nd) + corr2nd*cov2nd/cov1st)

}

if(cov1st < cov2nd){

firstSecond = cov2nd*(cov1st*(1 - corr2nd) + corr2nd*cov1st/cov2nd)

}

#figure out what proportion get just one dose

firstOnly = cov1st - firstSecond

secondOnly = cov2nd - firstSecond

#figure out what proportion get 0 doses

none = 1 - firstOnly - secondOnly - firstSecond

#multiply by age structure to get number of individuals in each age class who fall into each vaccine class

base = pop

firstOnly1 = firstOnly*base

secondOnly1 = secondOnly*base

firstSecond1 = firstSecond*base

none1 = none*base

#iterate through age classes

for(j in 1:length(pop)){

#immunize people eligible for the first dose (assume everyone older than them is immunized with the same proportion)

if(j == (target1st+1)){ #R indexes from 1 so we need to add 1 (for example, the 10th age class is 9 month olds)

firstOnly1[j:length(pop)] = (eff[j]+failure)*firstOnly1[j:length(pop)] #this is the susceptibles remaining after vaccination

firstSecond1[j:length(pop)] = (eff[j]+failure)*firstSecond1[j:length(pop)]

}

#immunize people eligible for the second dose (assume everyone older than them is immunized with the same proportion)

if(j == (target2nd+1)){

secondOnly1[j:length(pop)] = (eff[j] + failure)*secondOnly1[j:length(pop)]

firstSecond1[j:length(pop)] = (eff[j] + failure)*firstSecond1[j:length(pop)]

}

}

#find immune distribution

dists = pop - firstOnly1 - secondOnly1 - firstSecond1 - none1 + eff*pop #need to add back maternally immune individuals - they were immune at the time of vaccination

#find average immune distribution by proportion

distProps = dists/pop

#find average proportion immune

prop = sum(dists)/sum(pop)

result <- list(dists = dists, distProps = distProps, prop = prop)

return(result)

}

At equilibrium, this function returns the age distribution of immunity (dists). However, in a dynamical model, this function returns the trajectory of immunity for a specific birth cohort. For example, if age classes are monthly, then dists[j+1] is the status of dists[1] after j months have passed. The values passed to the function can be adjusted accordingly. In particular, eff can be varied to reflect a changing proportion of immune parents, and any change in vaccine failure of the second dose due to the recipient’s immune response to the first dose.
