# Supplementary material for: Correlation between measles vaccine doses: implications for the maintenance of elimination
Source: Epidemiol Infect. 2018 Feb 21;146(4):468–75. doi: 10.1017/S0950268817003077 (PMC5848754; doi:10.1017/S0950268817003077)
Supplement: Supplementary file 1 [file S0950268817003077sup001.zip › S0950268817003077sup001/Correlation_Supplement.2_10.2.2017.docx]

We can find similar equations for the situation when $v_{1}<v_{2}$. In this case, correlation is the proportion of the first dose administered non-independently to individuals who will later receive the second dose. When $v_{1}<v_{2}$, $p\left( MCV2|MCV1 \right)= v_{2}(1 - corr) + corr$ and ${p\left( MCV2 | \neg MCV1 \right)= v}_{2}(1 - corr) + corr(v_{2} - v_{1})$. Therefore, $v_{1} - v_{1}*v_{2}\left( 1 - corr \right)- v_{1}corr$ adults receive only the first dose, $v_{1}*v_{2} (1 - corr) + v_{1} corr$ adults receive both doses, and $\left( 1 - v_{1} \right)v_{2}(1 - corr)$ adults receive only the second dose. Again, the remainder are unvaccinated. In practice, second dose coverage almost never exceeds first dose coverage, though such a scenario could be envisioned in the event of a mandatory school entry dose; we include it for completeness.

The equilibrium proportion born with maternal immunity in this case is $p_{mT}=\left( 1 - p_{m(T-1)} w_{t_{1}}-p_{f} \right) *\left( v_{1} - v_{1}*v_{2}\left( 1 - corr \right)- v_{1}corr \right)+ \left( 1 - p_{m\left( T-1 \right)} w_{t_{2}}-p_{f} \right)* \left( \left( 1 - v_{1} \right)v_{2}(1 - corr) \right)+\left( 1-(p_{m\left( T-1 \right)}w_{t_{1}}+p_{f})(p_{m\left( T-1 \right)}w_{t_{2}}+p_{f}) \right) *(v_{1}*v_{2} (1 - corr) + v_{1} corr$).
